# Mogroside IIe Ameliorates Cardiomyopathy by Suppressing Cardiomyocyte Apoptosis in a Type 2 Diabetic Model

**DOI:** 10.3389/fphar.2021.650193

**Published:** 2021-05-03

**Authors:** Xin Cai, Lingmin He, Guoao Zhou, Shenghua Li, Xinghua Liao

**Affiliations:** ^1^ School of Life Science and Health, Wuhan University of Science and Technology, Wuhan, China; ^2^ School of Biotechnology and Food Engineering, Anyang Institute of Technology, Anyang, China

**Keywords:** MGE II, type 2 diabetic cardiomyopathy, apoptosis, SD rats, H9c2 cells

## Abstract

Mogroside II_e_ is primarily present in the unripe fruit of *Siraitia grosvenorii (Swingle) C. Jeffrey*, and it is the predominant saponin component. The purpose of this study was to investigate the effects of mogroside IIe (MGE IIe) on myocardial cell apoptosis in diabetic cardiomyopathy (DCM) rats by establishing a high-sugar and high-fat diet–induced model of type 2 diabetes (T2D) in SD rats and a homocysteine (Hcy)-induced apoptotic model in rat H9c2 cardiomyocytes. The results showed that MGE IIe decreased the levels of fasting blood glucose (FBG), total cholesterol (TC), triglyceride (TG), and low-density lipoprotein (LDL) levels, but increased the levels of high-density lipoprotein (HDL) in the SD rat model. Furthermore, MGE IIe decreased the levels of lactate dehydrogenase 2 (LDH2), creatine phosphokinase isoenzyme (CKMB), and creatine kinase (CK), and improved heart function. Additionally, MGE IIe inhibited the secretion of interleukin-1 (IL-1), IL-6, and tumor necrosis factor-α (TNF-α), improved myocardial morphology, and reduced myocardial apoptosis in the SD rat model. Furthermore, MGE IIe inhibited the mRNA and protein expression of active-caspase-3, -8, -9, -12, and Bax and Cyt-C, and promoted the mRNA and protein expression of Bcl-2 in the SD rat model. Furthermore, MGE IIe suppressed homocysteine-induced apoptosis of H9c2 cells by inhibiting the activity of caspases-3, -8, -9, and -12. In conclusion, MGE IIe inhibits the apoptotic pathway, thereby relieving DCM *in vivo* and *in vitro*.

## Introduction

Diabetic cardiomyopathy (DCM) generally refers to diabetes-related myocardial dysfunction in the absence of other apparent causes. Moreover, DCM is one of the most long-lasting cardiovascular diseases and has generated much interest in recent years (Shetty et al., 2018; Zheng et al., 2018). DCM pathogenesis is complex and involves a wide range of factors, including abnormal insulin secretion, glucose, and lipid metabolism disorders, as well as inflammatory responses (Xing et al., 2019). Clinical manifestations of DCM include metabolic disorders, myocardial cell abnormalities, and microvascular lesions. These manifestations further myocardial cell apoptosis and myocardial fibrosis, and lead to ventricular wall stiffness and cardiac function damage, resulting in heart failure and eventually death in severe cases (Song et al., 2017; Yu et al., 2017).

Apoptosis is an active and orderly process of programmed cell death, which is regulated by multiple genes and proteases (Wang et al., 2018). DCM manifests as metabolic disorders and cardiomyocytes abnormalities that are related to cardiomyocyte apoptosis (Cuomo et al., 2019). Therefore, it is necessary to explore the effects of apoptosis on myocardial injury in DCM and identify drugs that inhibit myocardial injury in patients with DCM.

Mogroside (MGE) is an important monomer extracted from *Siraitia grosvenorii (Swingle) C. Jeffrey*. It belongs to the triterpene compounds, including MGE Ⅱe, Ⅲ, Ⅲe, Ⅳ, Ⅴ, and Ⅵ as well as serotonin I. Among them, MGE Ⅱe is primarily present in the unripe fruit of *Siraitia grosvenorii (Swingle) C. Jeffrey* (Xiao et al., 2020). Studies have shown that MGE reduces blood glucose and lipid levels in diabetic mice, eliminates reactive oxygen species *in vitro* (Zhang et al., 2018; Liu et al., 2019; Zhang et al., 2020), and alleviates high glucose-induced inflammation and oxidative stress in podocytes *in vitro* (Xue et al., 2020). However, whether MGE IIe can improve T2D cardiomyopathy by inhibiting cardiomyocyte apoptosis is unclear.

In this study, we established a DCM rat model to study whether myocardial injury is related to apoptosis of myocardial cells and whether inhibition of apoptosis was a cause of myocardial injury. Determining whether MGE IIe therapy can improve apoptosis in myocardial cells and alleviate myocardial injury would provide a scientific and theoretical basis for DCM treatment using MGE IIe.

## Materials and Methods

### Reagents and Antibodies

The MGE IIe (purity 98%) was purchased from Honghe Qianshan Bioengineering Co., Ltd. (Honghe, China). Streptozocin (STZ) and rosiglitazone were obtained from Multi Sciences Biotech Co. Ltd. (Hangzhou, China). Antibodies against *ß*-actin, caspase-3, -8, -9, -12, Bax, Cyt-C, and Bcl-2 were obtained from Cell Signaling Technology (Danvers, MA, United States). ELISA kits for the detection of IL-1, IL-6, TNF-α, CK-MB, HDL, and LDL were purchased from Thermo Fisher Scientific (Waltham, MA, United States). The mRNA extraction kit, reverse transcription kit, PCR mixture, and Masson staining kit were purchased from Tiangen Biochemical Technology Co., Ltd. (Beijing, China). The Cell Counting Kit-8 (CCK-8) was purchased from Dojindo Molecular Technologies (Kumamoto, Japan).

### Animal Model

Adult male Sprague–Dawley (SD) rats (180–200 g), obtained from the Experimental Animal Center of the Wuhan University of Science and Technology (China), were kept under standardized conditions as follows: room temperature, 22 ± 2°C; relative humidity, 45–55%; and a 12-h light/dark cycle in the animal facility with free access to food and water.

Two weeks after completing the adaptive rat feeding, the control group continued to receive a normal diet. The remaining rats were fed a high-fat, high-sugar (HFHS) diet (15% lard, 30% sucrose, 2% cholesterol, 1% sodium cholate, 5% protein powder, and 47% regular diet). After 8 weeks of feeding, the rats were intraperitoneally injected with 35 mg/kg STZ to induce a model of T2D. Animals with fasting blood glucose (FBG) levels higher than 16.7 mmol/L three days after the STZ injection constituted successful T2D models. The rats were then orally administered MGE IIe at a dose of 30 (low-dose, LD) or 60 mg/kg/d (high dose, HD), and rosiglitazone at 5 mg/kg/d for 8 weeks.

### Cell Culture

Rat H9c2 cardiomyocytes were obtained from the American Type Culture Collection (ATCC). The cells were cultured at 37°C in a 5% CO_2_ incubator in Rosewell Park Memorial Institute 1,640 (RPMI1640) medium supplemented with 10% fetal bovine serum. The cells were used for incubation after differentiation with 1% serum accompanied by 1 μM all-trans retinoic acid for a few days. Next, the cells were pretreated with MGE IIe for 4 h and then induced with 1.5 mM homocysteine (Hcy) for 24 h.

### Biochemical Index Detection

The FBG levels were measured using a glucose meter (Accu-Check Performa, Mannheim, Germany). Triglyceride (TG) and total cholesterol (TC) levels were measured using an automated biochemical analyzer. The levels of insulin, IL-1, IL-6, TNF-α, low-density lipoprotein (LDL), high-density lipoprotein (HDL), and creatine phosphokinase isoenzyme (CKMB)were detected using ELISA kits (Bai et al., 2019). The activity of creatine kinase (CK) was detected according to Hughes (Hughes, 1962). Samples (approximately 1 μg of protein) were incubated in a medium consisting of 50 mM Tris buffer, pH 7.5, 7.5 mM MgSO4, 7.1 mM phosphocreatine, and 3.2 mM ADP for 10 min at 37°C when the reaction was stopped by the addition of 20 μL of 50 mM P-hydroxymercuribenzoic acid. The colorimetric reaction was carried out by adding 20% *a*-naphthol and 20% diacetyl and was read after 20 min of incubation (37°C) at 540 nm. A calibration curve was constructed using creatine that was subjected to the same procedure. The levels of malondialdehyde (MDA), superoxide dismutase (SOD), and glutathione peroxidase (GSH) were determined using the thiobarbituric acid (TBA), hydroxylamine, and microplate methods, respectively, following the manufacturer’s instructions.

### Real-Time Polymerase Chain Reaction

Total RNA was extracted from the heart tissues using a total RNA extractor (TRIzol) kit. Then the cDNA for each RNA sample was reverse transcribed using an rtase cDNA kit according to the manufacturer’s instructions. Real-time PCR was performed using SYBR Premix EX Tap 2× kit and a PT-PCR system. The reaction conditions were as follows: 95°C for 30 s, 95°C for 5 s, 60°C for 30 s, followed by 30 cycles at 72°C for 10 min according to the provided instructions. The primer sequences are listed in Table 1. Ribosomal 18S is an internal standard for the quantitative comparison of mRNA levels (Goidin et al., 2001). The relative expression of each gene was calculated using the 2^−ΔΔCt^ method (Livak and Schmittgen, 2001).

**TABLE 1:** Primer parameters.

Name	Sequence	Tm (°C)	Amplicon length
Caspase-3	F: ACAGGAGAGCAGGGATTT	60	141
	R: CAC​CAT​TTC​AGT​AGC​AGG​A		
Caspase-8	F: CGAGAAGGGAGGACAGAG	60	127
	R: ACA​CCA​CAT​AGA​GGC​AGA​AG		
Caspase-9	F: CCA​ACA​AAA​CTA​ATC​CCA​AG	60	123
	R: CCA​AAC​CCT​ATC​TCC​TGA​A		
Caspase-12	F: CTG​CTT​GGC​TCT​TCT​CTT​T	60	89
	R: CTTGTTTGCGATGTCTCC		
Bax	F: GAT​TAC​GTG​AGG​AGA​TAG​A	60	105
	R: ATG​CCA​CAT​AGA​CGC​AGA​G		
Bcl-2	F: AGCGACAGCAGGGATTAT	60	99
	R: CCAGTTTCGGTAGCAGGA		
Cyc-C	F: GGAAAGCAAAGACCACCT	60	150
	R: GTTCAAAGCAGGAGAGCA		

### Hematoxylin–Eosin, Masson’s Trichrome, Immunohistochemical, and Terminal Deoxynucleotidyl Transferase dUTP Nick End Labeling (TUNEL) Staining

Small pieces of myocardium tissue were fixed with 10% paraformaldehyde. The samples were then washed with tap water and subjected to a series of processing steps, including dehydration, rendering transparent, paraffin embedding, and sectioning for hematoxylin-eosin (HE) or Masson’s trichrome staining. The samples were then stained with HE for 10 min, differentiated with ethanolic hydrochloric acid for 20 s, and rinsed with water for 10 min. After dehydration with ethanol, the samples were rendered transparent with xylene and sealed with a neutral gel. For immunohistochemical detection, paraffin sections were retrieved with citric acid and incubated with the primary antibodies at 4°C overnight. The secondary antibody was then added and samples were incubated at 37°C for 15 min. For TUNEL staining, after dewaxing the liver paraffin sections, proteinase K was added to the sections and incubated for 30 min, followed by incubation with a mixture of TdT enzyme and biotin-dUTP (TdT:Biotin-dUTP = 1:9) was added and incubated for 1 h at 37°C. The converter-AP solution was then added to the sections which were then incubated at 37°C for 20 min. Finally, BCIP/NBT and nucleus red solutions were used in a color reaction and AEC aqueous sealing tablets were used to seal the sections.

### Western Blotting

Total proteins were extracted using radioimmunoprecipitation assay (RIPA) lysis buffer containing PMSF, and quantified using BCA. The final protein concentration was adjusted to 5 μg/μL. Protein samples (40 μg) were then separated using 8% sodium dodecyl sulfate polyacrylamide gel electrophoresis (SDS-PAGE), transferred to polyvinylidene fluoride (PVDF) membranes, and blocked with 5% milk for 120 min at room temperature. Then, the membranes were incubated with primary antibodies (1:1,000 dilution) against active-caspase-3, -8, -9, -12, Bax, Bcl-2, and Cyt-C overnight at 4°C. Horseradish peroxidase (HRP)-conjugated anti-rabbit or mouse IgG (1:3,000 dilution) served as secondary antibodies. The protein bands in the membranes were visualized using enhanced chemiluminescence (ECL) detection system. Signal intensity was quantified using ImageJ software.

### CCK-8 Assay

H9c2 cells (1 × 10^3^ cells per well) were seeded in a 96-well plate, and treated with different concentrations of Hcy or MGE IIe. The viability of cells was determined at every 24 h. Before the test, 10 μL CCK-8 (Dojindo Molecular Technologies, Kumamoto, Japan) was added into each well and incubated at 37°C for 2 h. Then the OD values were measured at 450 nm using a spectrophotometer (BioTek, Winooski, VT, United States).

### Flow Cytometry

H9c2 cells were pretreated with 20, 50, and 100 μM MGE IIe for 4 h, and then induced with 1.5 mM Hcy for 24 h. Next, the adherent cells were collected in a 15 ml centrifuge tube and centrifuged at 1,000 rpm for 5 min. The supernatant was discarded, and 500 μL of PBS was added to the tube to resuspend the cells. A total of 10 μL PI and 5 μL FITC were added to the tubes and the tubes were incubated in the dark. The cells were finally subjected to flow cytometric analysis, according to the manufacturer’s instructions.

### Statistical Analysis

The statistical analyses were conducted using SPSS software (version 13.0). The data were expressed as the mean ± standard error of the mean (SEM). One-way ANOVA was followed by post hoc tests for comparisons among groups, where *p* < 0.05 indicated that the difference was statistically significant.

## Results

### Diabetic Cardiomyopathy Animal Model

The FBG and lipid factors were analyzed to determine whether the DCM model had been successfully established. The results showed that the blood sugar, TC, TG, and LDL levels had significantly increased, whereas the HDL level had markedly decreased in the DCM model group compared to those in the control group (Supplementary Data A,B). Subsequently, rat cardiac function was evaluated. The results showed that the ratio of heart weight to body weight and biochemical factors LDH1, CKMB, and CK were markedly increased in the DCM model group compared to those in the control group (Supplementary Data C,D). In addition, pathological changes in the heart were evaluated by HE staining. The results showed abnormal cardiomyocyte arrangement in the model group, with small gaps between cardiomyocytes (Supplementary Data E). Furthermore, the fibrotic changes in the myocardium were detected using Masson's trichrome staining. The results showed that the collagen fiber deposition in rat myocardial tissue was significantly increased in the model group compared to that in the control group (Supplementary Data F). The above results indicate that the DCM model had been successfully established.

### Mogroside IIe Improves Cardiac Function and Inhibits Apoptosis of Cardiomyocytes

**FIGURE 1:**
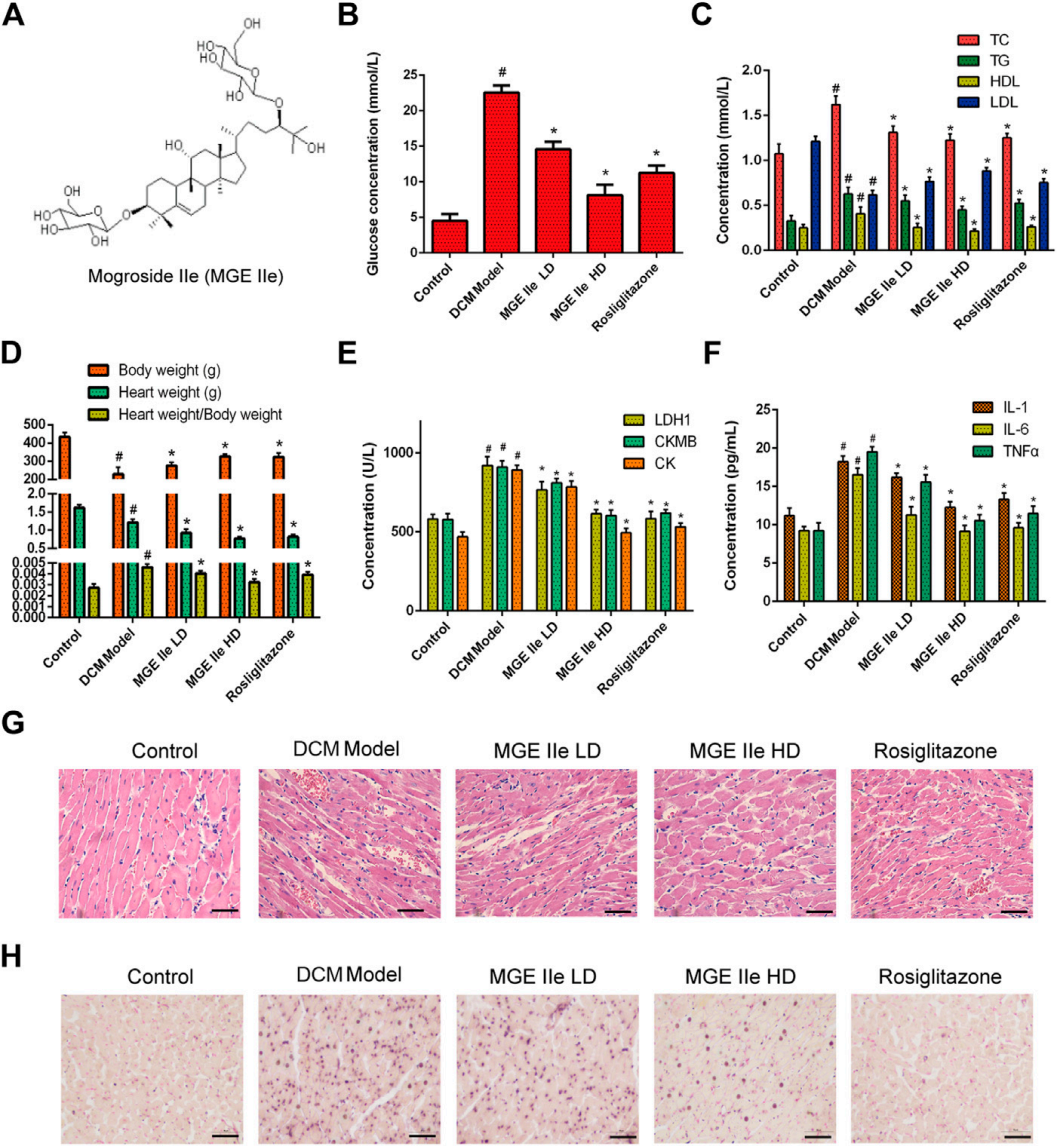
MGE IIe slows down the damage to cardiomyocytes in the DCM model. **(A)** The structural formula of MGE IIe. **(B)** MGE IIe decreased the serum glucose concentration. **(C)** MGE IIe inhibited lipid accumulation. **(D)** MGE IIe decreased the ratio of heart to body weight. **(E)** MGE IIe improved cardiac function. **(F)** MGE IIe inhibited the secretion of inflammatory cytokines. **(G)** MGE IIe improved cardiomyocyte damage as revealed by HE staining. **(H)** MGE IIe reduced cardiomyocyte apoptosis as revealed by TUNEL staining assay. Bar = 50 µm. One-way ANOVA was followed by post hoc tests for comparisons among groups. The values shown represent the mean ± standard error of the mean (SEM) of the data from three independent experiments. ^#^ *p* < 0.05 (compared to the control group alone); ^*^ *p* < 0.05 (compared to the model group alone).

**FIGURE 2:**
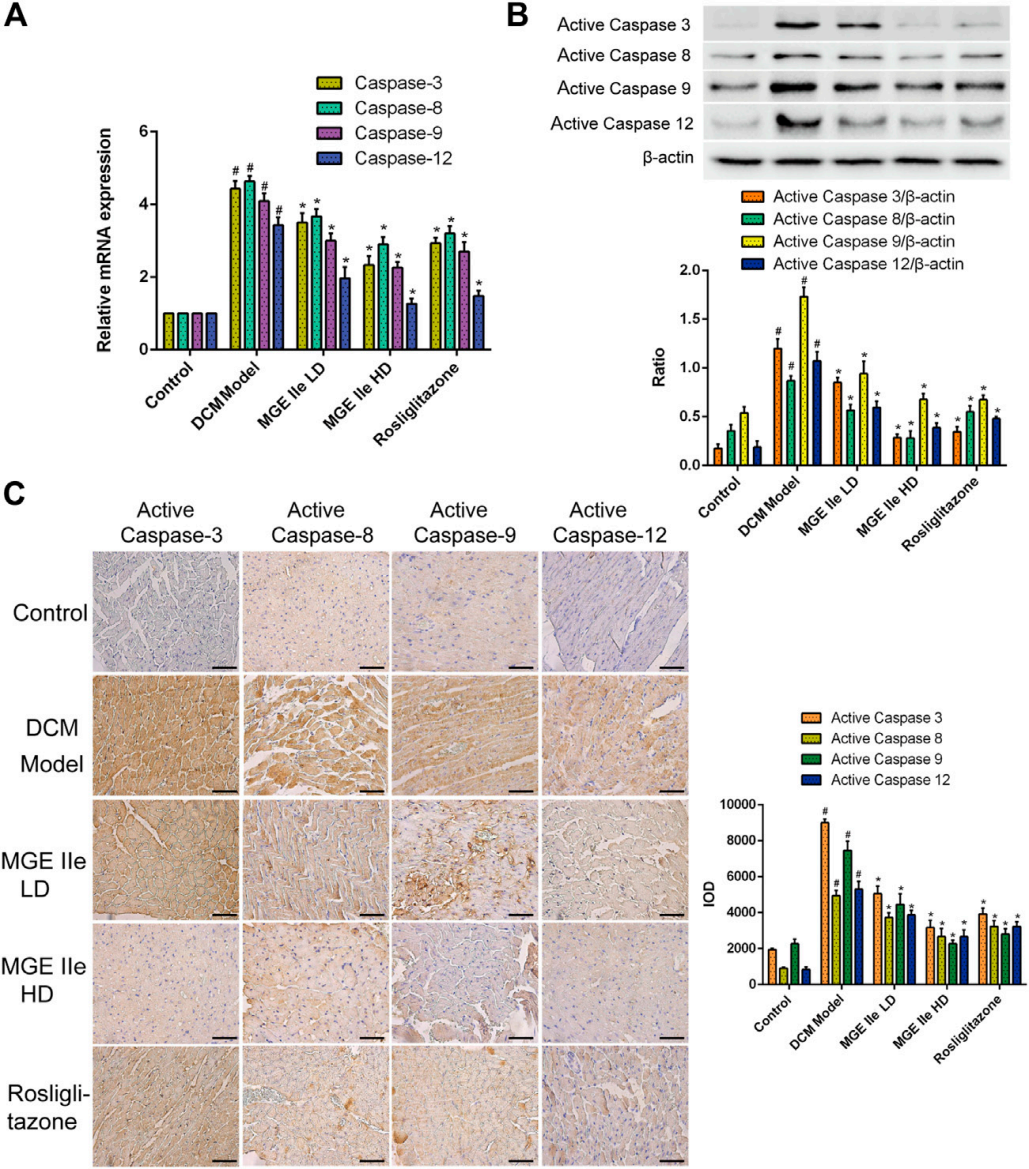
MGE IIe inhibits the expression of apoptotic factors of the caspase family. **(A)** MGE IIe inhibited mRNA expression of caspase-3, -8, -9, and -12 as assessed by qPCR assay. **(B)** MGE IIe suppressed the activity of caspase-3, -8, -9, and -12 as revealed by western blot assay. **(C)** MGE IIe suppressed the activity of caspase-3, -8, -9, and -12 as revealed by IHC assay. Bar = 50 µm. One-way ANOVA was followed by post hoc tests for comparisons among groups. The values shown represent the mean ± standard error of the mean (SEM) of the data from three independent experiments. ^#^ *p* < 0.05 (compared to the control group alone); ^*^ *p* < 0.05 (compared to the model group alone).

The effect of MGE IIe (Figure 1A) in the DCM model was examined. Rosiglitazone, an anti-diabetic drug, was used as a positive control. The results showed that MGE IIe decreased the levels of glucose, TG, TC, and LDL, and increased the level of HDL in a dose-dependent manner (Figures 1B,C). Furthermore, MGE IIe improved heart function by reducing the ratio of heart weight to body weight and the levels of LDH1, CKMB, and CK in a dose-dependent manner (Figures 1D,E). In addition, MGE IIe suppressed the secretion of the inflammatory factors IL6, IL1, and TNF-α (Figure 1F). Furthermore, HE staining results showed that MGE IIe gradually normalized the arrangement of cardiomyocytes in a dose-dependent manner (Figure 2G). TUNEL staining results showed that MGE IIe inhibited myocardial cell apoptosis in the DCM rat model in a dose-dependent manner (Figure 1H). The above results showed that MGE IIe improved cardiac function and inhibited cardiomyocyte apoptosis in a dose-dependent manner.

### Mogroside IIe Inhibits the Apoptotic Signaling Pathway

**FIGURE 3:**
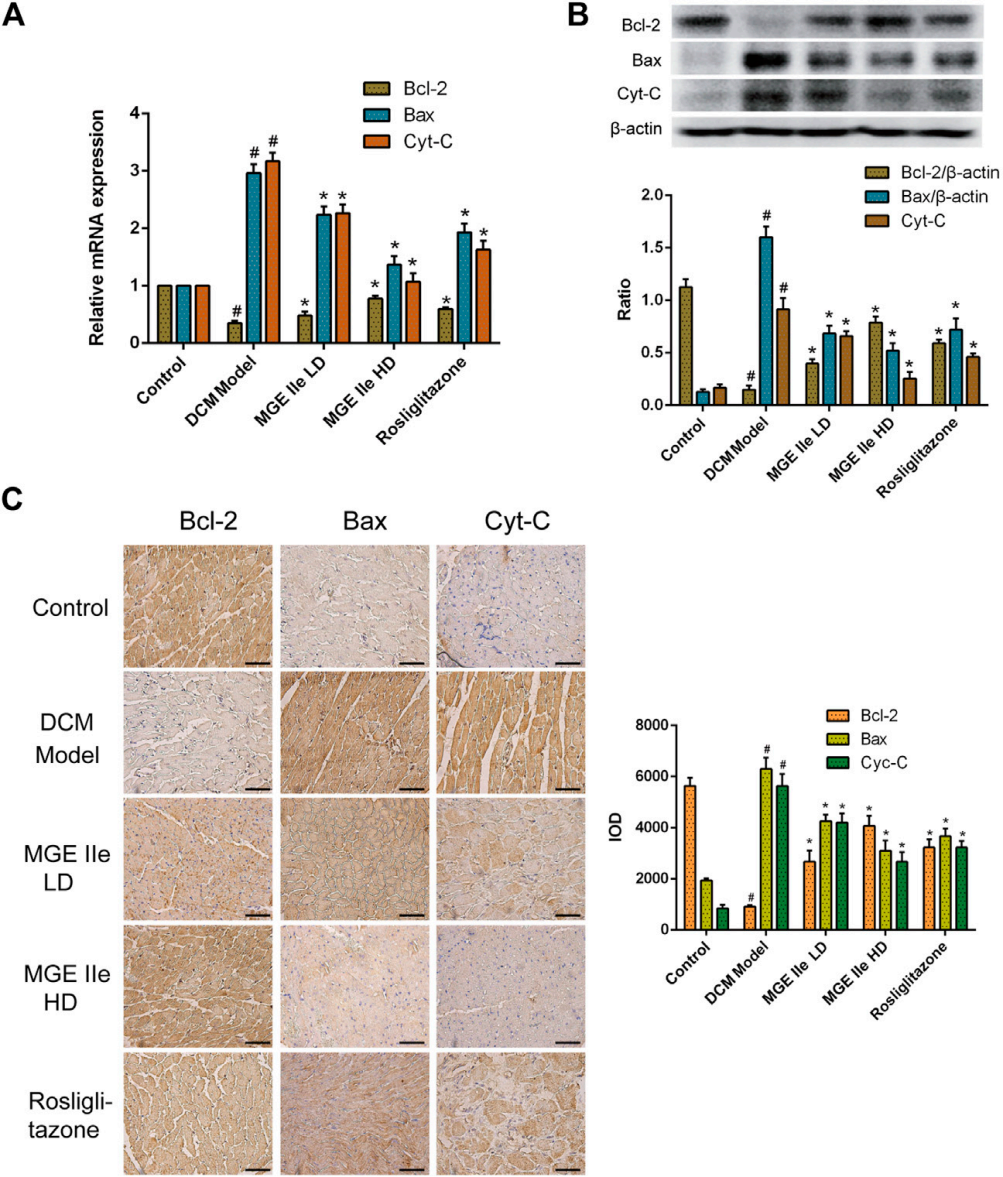
MGE IIe inhibits the expression of apoptotic factors Bax and Cyt-C and promotes the expression of Bcl-2. **(A)** MGE IIe inhibited the mRNA expression of Bax and Cyt-C, and promoted the mRNA expression of Bcl-2 as revealed by qPCR assay. **(B)** MGE IIe suppressed the protein expression of Bax and Cyt-C and promoted the protein expression of Bcl-2 as revealed by western blot assay. **(C)** MGE IIe suppressed the protein expression of Bax and Cyt-C and promoted the protein expression of Bcl-2 as revealed by IHC assay. Bar = 50 µm. One-way ANOVA was followed by post hoc tests for comparisons among groups. The values shown represent the mean ± standard error of the mean (SEM) of the data from three independent experiments. ^#^ *p* < 0.05 (compared to the control group alone); ^*^ *p* < 0.05 (compared to the model group alone).

Based on these results, we further analyzed the activity of apoptosis-related factors. First, the mRNA expression levels of caspase-3, -8, -9, and -12 were detected. The results showed that MGE IIe inhibited the mRNA expression of caspase-3, -8, -9, and -12 in a dose-dependent manner (Figure 2A). Furthermore, the effect of MGE IIe on caspase activity was examined by western blotting and IHC staining. The results showed that MGE IIe inhibited the activity of caspase-3, -8, -9, and -12 in a dose-dependent manner (Figures 2B,C). Next, additional factors, including Bax, Bcl-2, and Cyt-C that are also closely related to apoptosis, were analyzed. The results showed that MGE IIe decreased the mRNA and protein expressions of Bax and Cyt-C, whereas it promoted the mRNA and protein expressions of Bcl-2 in a dose-dependent manner (Figure 3). These results showed that MGE IIe inhibited apoptosis of myocardial cells in a dose-dependent manner in the DCM model.

### Mogroside IIe Suppresses the Hcy-Induced Apoptosis of H9c2 Cells

**FIGURE 4:**
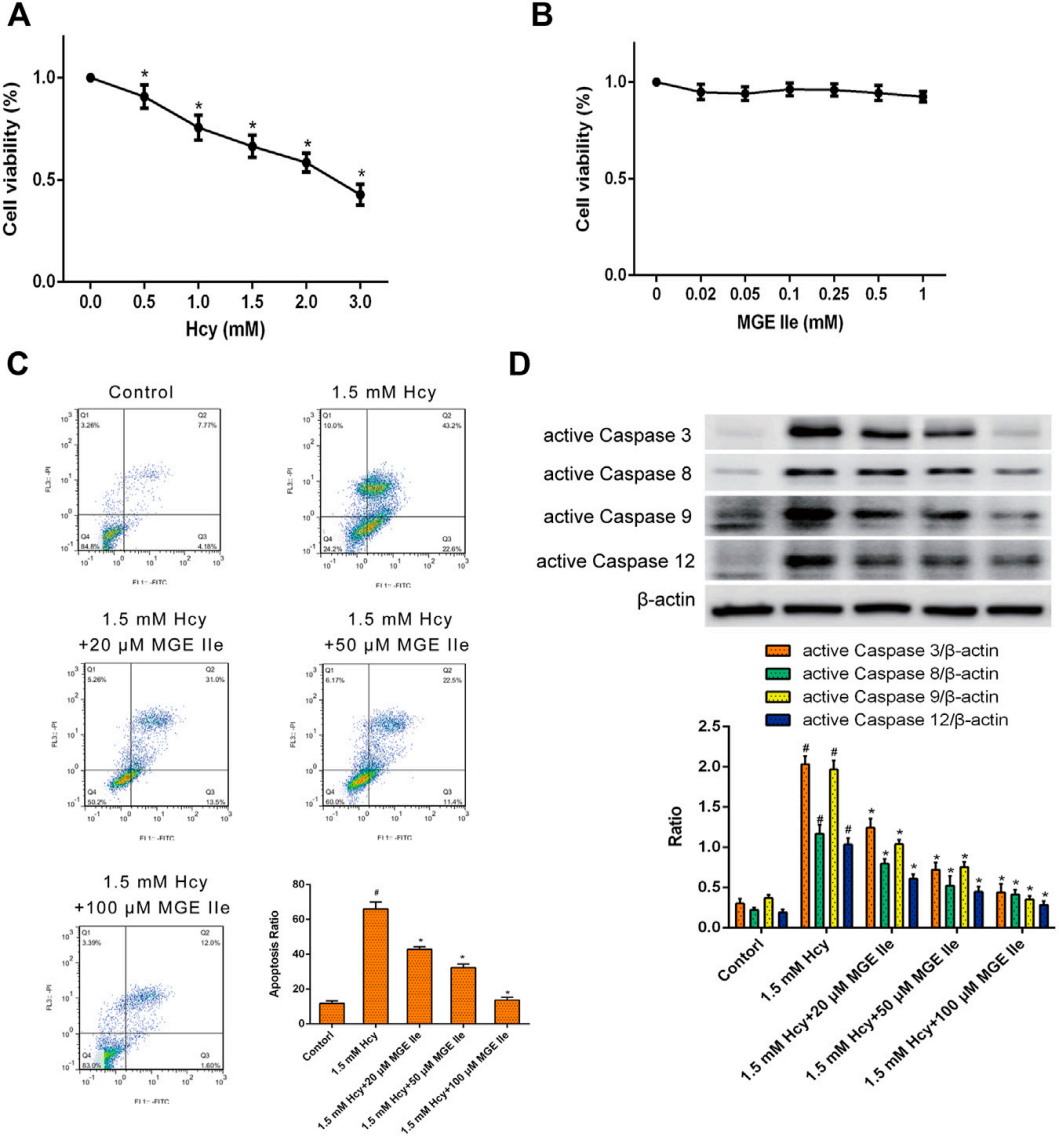
MGE IIe inhibits the apoptosis of H9c2 cells induced by Hcy. **(A)** The effect of Hcy on H9c2 cell viability. **(B)** The effect of MGE IIe on the H9c2 cell viability. **(C)** The effect of MGE IIe on the Hcy-induced H9c2 cells apoptosis model by flow cytometry. **(D)** The effect of Hcy on the activity of caspase-3, -8, -9, and -12 in H9c2 cells. One-way ANOVA was followed by post hoc tests for comparisons among groups. The values shown represent the mean ± standard error of the mean (SEM) of the data from three independent experiments. ^#^ *p* < 0.05 (compared to the control group alone); *p < 0.05 (compared to the model group alone).

The effect of MGE IIe on the apoptosis of H9c2 myocardial cells *in vitro* was further examined*.* H9c2 cells were treated with different concentrations of Hcy or MGE IIe. The CCK8 assay results showed that Hcy inhibited the activity of H9c2 cells in a dose-dependent manner (Figure 4A). In contrast, MGE IIe had no significant influence on the viability of H9c2 cells (Figure 4B). Furthermore, flow cytometric analysis revealed that H9c2 cell apoptosis was significantly induced by Hcy, whereas MGE IIe inhibited the Hcy-induced apoptosis of H9c2 cells in a dose-dependent manner (Figure 4C). In addition, MGE IIe downregulated the expression of active caspase-3, -8, -9, -12, and Bax and Cyt-C, and upregulated the expression of Bcl-2 in a dose-dependent manner (Figure 4D). The above results showed that MGE IIe inhibited Hcy-induced apoptosis in H9c2 cells.

## Discussion

Injury to tissues and organ functions are the main complications associated with the occurrence and development of diabetes. An SD mouse model of T2D was generated using a high-sugar and high-fat diet combined with low-dose STZ injection (Gu et al., 2017). This animal model has been previously used to study various aspects related to T2D, such as symptoms, progression, and complications (Barrière et al., 2018). The model has also been used in pharmacological experiments to identify drugs that are more effective (Liu et al., 2018).

After successfully establishing the T2D model using this method, we found that rats in the HFHS treatment group had higher glucose, TC, TG, and LDL levels and lower HDL levels compared to rats in the normal diet group (Supplementary Data A,B). This validated that the T2D model was successfully established. We further examined whether these animal models exhibited T2D-induced cardiomyopathy. The ratio of heart weight to body weight and the levels of LDH1, HDL, CKMB, and CK were increased in the model group (Supplementary Data C,D). In addition, several empty structures and collagen fibrous deposition were present around the cardiomyocytes and the distance between cells was increased the model group (Supplementary Data E,F). These findings suggested that the T2D rats in this study sustained myocardial damage. Therefore, the *in vivo* DCM model was successfully established. We also found that cardiomyocyte apoptosis was significantly increased in the DCM model. Furthermore, an Hcy-induced myocardial cell apoptosis was established *in vitro*. This model is widely used to study cardiomyocyte apoptosis (Zhang et al., 2017; Aminzadeh & Mehrzadi, 2018; Chen et al., 2020).

Cardiomyocyte apoptosis plays a key role in the mechanism for myocardial injury in diabetes mellitus. Studies have shown that the caspase family plays an important role in apoptosis execution (Wang et al., 2019). Caspase-3 constitutes the central molecule in apoptosis and is involved in three main pathways: the exogenous death receptor pathway, endogenous mitochondrial pathway, and endoplasmic reticulum pathway (Espinosa-Oliva et al., 2019). The exogenous death receptor pathway is triggered by a transmembrane protein reaction caused by TNF-α, and mainly involves caspase-8, a pro-apoptotic factor (DeLaney et al., 2019). The mitochondria-mediated apoptotic pathway regulates the positive and negative regulators of apoptosis including Bcl-2 and Bax. Its main mechanism is to regulate the release of Cyt-C by coordinating mitochondrial membrane permeability (Xiong et al., 2014; Wei et al., 2018). In addition, Ca^2+^ content in the endoplasmic reticulum is the highest, and it mainly regulates the concentration of Ca^2+^ to coordinate the signal transduction of cells. When the endoplasmic reticulum is severely damaged, apoptosis is initiated by the activation of caspase-12 and Bax, and caspase-12 is transferred to the factor caspase-9 (Jong et al., 2017). Activated caspase-9 stimulates caspase-3 and causes apoptosis (Honda et al., 2007). The present study found that the above proteins reflecting apoptosis were altered in DCM and Hcy-induced apoptotic models of cardiomyocytes. Therefore, it is necessary to identify drugs against myocardial apoptosis to alleviate the occurrence of DCM.

*Siraitia grosvenorii (Swingle) C. Jeffrey* (luo-han-guo or monk fruit, NCBI Taxonomy ID: 190,515) is a herbaceous perennial native to southern China, and has been used for centuries as a natural sweetener and traditional Chinese medicine for the treatment of dry cough, lung congestion, sore throats and colds, as well as constipation and intestinal ailments (Li et al., 2014).

MGE IIe is the main component extracted from *Siraitia grosvenorii (Swingle) C. Jeffrey*, and is used as both medicine and food. It also has anti-inflammatory and antioxidant properties.

The present study found that MGE IIe gradually decreased the heart-body ratio (*p* < 0.05). It also downregulated the levels of glucose, TG, TC, LDL, CLDH1, CKMB, CK, IL-1, IL-6, and TNF-α, and upregulated HDL levels in a dose-dependent manner (*p* < 0.05). The results of pathological staining showed that the disorder of cardiac cells was improved and the intercellular space was reduced, whereas the apoptosis of cardiomyocytes gradually decreased following treatment with MGE IIe. Further analysis of the apoptotic signaling pathways showed that MGE IIe regulates the expression of mRNA and the protein activity of caspase-3, -8, -9, -12, and Bcl-2, Bax, and Cyt-C (*p* < 0.05). A high dose of MGE IIe was more effective than rosiglitazone. Therefore, MGE IIe can be used as an additive to produce food and health care products for glucose-reducing and immunity enhancement, and can also be used in combination with antidiabetic drugs where it has play an adjunctive therapeutic role.

In conclusion, our data suggest that MGE IIe may alleviate DCM by inhibiting cardiomyocyte apoptosis *in vitro* and *in vivo*.

## Data Availability

The raw data supporting the conclusions of this article will be made available by the authors, without undue reservation.
