# Supplementary figures and images for: Mogroside IIe Ameliorates Cardiomyopathy by Suppressing Cardiomyocyte Apoptosis in a Type 2 Diabetic Model
Source: Front Pharmacol. 2021 May 3;12:650193. doi: 10.3389/fphar.2021.650193 (PMC8128068; doi:10.3389/fphar.2021.650193)

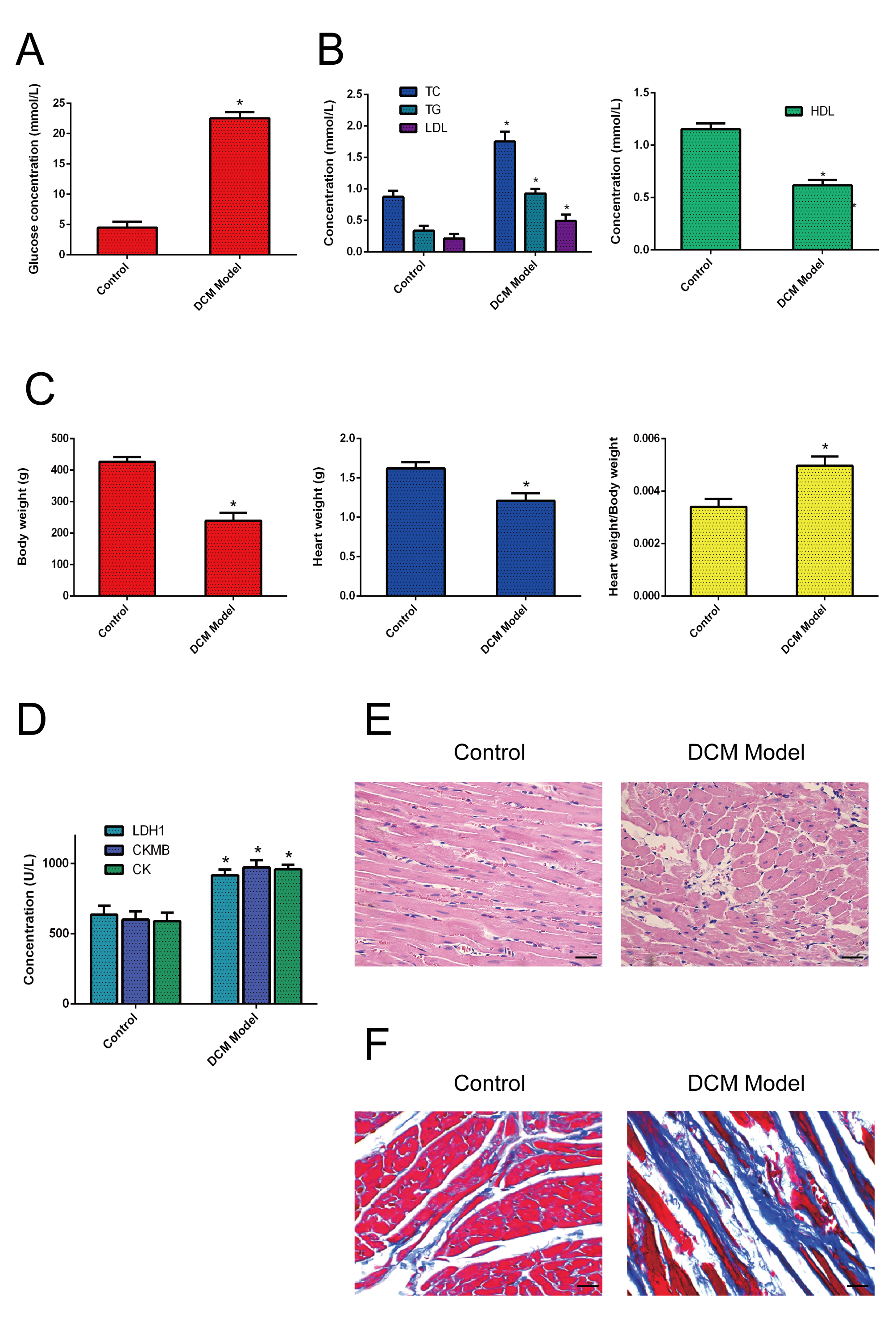

Supplement: Supplementary file 1 [file Image1.TIF]
